# Structural analysis of the GPI glycan

**DOI:** 10.1371/journal.pone.0257435

**Published:** 2021-09-16

**Authors:** Miyako Nakano, Susana Sabido-Bozo, Kouta Okazaki, Auxiliadora Aguilera-Romero, Sofia Rodriguez-Gallardo, Alejandro Cortes-Gomez, Sergio Lopez, Atsuko Ikeda, Kouichi Funato, Manuel Muñiz

**Affiliations:** 1 Graduate School of Integrated Sciences for Life, Hiroshima University, Higashi-Hiroshima, Hiroshima, Japan; 2 Facultad de Biologia, Departamento de Biologia Celular, Hospital Universitario Virgen del Rocio/CSIC/Universidad de Sevilla, Universidad de Sevilla e Instituto de Biomedicina de Sevilla (IBiS), Sevilla, Spain; University of Nebraska Medical Center, UNITED STATES

## Abstract

Glycosylphosphatidylinositol (GPI) anchoring of proteins is an essential post-translational modification in all eukaryotes that occurs at the endoplasmic reticulum (ER) and serves to deliver GPI-anchored proteins (GPI-APs) to the cell surface where they play a wide variety of vital physiological roles. This paper describes a specialized method for purification and structural analysis of the GPI glycan of individual GPI-APs in yeast. The protocol involves the expression of a specific GPI-AP tagged with GFP, enzymatic release from the cellular membrane fraction, immunopurification, separation by electrophoresis and analysis of the peptides bearing GPI glycans by mass spectrometry after trypsin digestion. We used specifically this protocol to address the structural remodeling that undergoes the GPI glycan of a specific GPI-AP during its transport to the cell surface. This method can be also applied to investigate the GPI-AP biosynthetic pathway and to directly confirm predicted GPI-anchoring of individual proteins.

## Introduction

In eukaryotic cells, many cell surface proteins are attached by the glycolipid glycosylphosphatidylinositol (GPI) to the external leaflet of the plasma membrane where they play diverse vital physiological roles including enzymes, receptors, and adhesion molecules [1,2]. GPI anchoring to proteins occurs as a conserved post-translational modification in the endoplasmic reticulum (ER), where the GPI has been previously synthesized by a series of sequential reactions involving more than 20 genes [3]. The core structure of the GPI anchor precursor consists of a phospholipid moiety acyl-phosphatidylinositol (acyl-PI) and a glycan backbone made of a glucosamine (GlcN) and four mannoses (Man), in which Man1, Man2, and Man3 have an ethanolamine phosphate (EtNP) side-branch. Once the GPI anchor has been made, the C-terminal of a newly synthesized integral protein is attached to the amine group of the bridging EtNP on Man3 in a reaction of transamidation by the GPI transamidase complex. After GPI attachment to the protein, the acyl PI is deacylated first and then the glycan part of the GPI anchor (GPI glycan) undergoes a structural remodeling during GPI-anchored protein (GPI-AP) transport to the cell surface, which is important for GPI-AP function and trafficking [3]. There is variability in the remodeling of the GPI glycan dependent upon proteins and species.

In our study, we were interested to determine how is remodeled the GPI glycan of the specific GPI-AP Gas1 (UniProt accession number: P22146) in the yeast *Saccharomyces cerevisiae* [4]. Earlier analysis methods to elucidate fully the structure of a GPI glycan in yeast were based on conventional carbohydrate chemistry, which is a lengthy task that requires large amounts of native protein and specialized techniques including radiolabeling and nuclear magnetic resonance [5]. Instead, we used a protocol based on a mass spectrometry approach, that enables a GPI structure to be elucidated from an immunopurified sample of the protein of interest separated on a SDS-polyacrylamide gel electrophoresis (SDS-PAGE) gel [6]. By using this method, we could analyze the structure of the remodeled GPI glycan of Gas1 tagged with GFP [7]. Wild type cells expressing Gas1-GFP were mechanically lysed with glass beads to subsequently obtain a cellular membrane fraction by differential centrifugation. Next, Gas1-GFP was released from the membranes upon enzymatic treatment with PI-specific phospholipase C (PI-PLC). PI-PLC specifically cleavages the lipid moiety of the non-inositol-acylated GPI anchor, releasing the protein in soluble form linked to the GPI glycan [8]. Because this enzymatic reaction is highly specific for GPI-APs, PI-PLC is an extremely useful tool to identify and characterize GPI-APs. Indeed, this method serves to experimentally confirm the GPI anchoring of many proteins that were only predicted to receive the GPI anchor by sequence comparisons [9]. Once Gas1-GFP is enzymatically removed from the cellular membranes by PI-PLC treatment, it is immunoprecipitated from the supernatant with anti-GFP antibodies. The purified Gas1-GFP was then separated by SDS-PAGE and stained with Coomassie Brilliant Blue. The stained band of Gas1-GFP was excised from the gel and then was subjected to in-gel digestion with trypsin after alkylation with iodoacetamide and reduction with dithiothreitol. Tryptic peptides and the peptides carrying GPI glycan were extracted, dried and analyzed by liquid chromatography–mass spectrometry (LC-MS). Sample preparation of peptides containing GPI glycan for LC-MS analysis was carried out according to the protocol for protein identification as described by Bhunchoth et al. [10] and the protocol for the N-glycan analysis on glycopeptide as described by Takahashi et al. [11].

For LC-MS analysis, the dried peptides were dissolved with water, injected into LC and separated using an Octadecyl-silica (ODS) column under specific gradient conditions. It is very important to dissolve the dried peptides with water, not with theoretical mobile phase containing formic acid for LC separation. Otherwise, the peptides carrying GPI glycan will be eluted with sodium formate polymer in the pass-through fraction, and it will be hard to detect the peptides carrying GPI glycan in the case of very short peptide. The eluate was introduced continuously into an electrospray ionization (ESI) source, and tryptic peptides and the peptides carrying GPI glycan were analyzed by LTQ Orbitrap XL (Thermo Fisher Scientific). MS data were obtained in positive ion mode over the mass range m/z 300 to m/z 3000 (resolution: 60,000, mass accuracy: 10 ppm) and MS/MS data were obtained by ion trap in LTQ Orbitrap XL.

## Materials and methods

The protocol described in this peer-reviewed article is published on protocols.io dx.doi.org/10.17504/protocols.io.bxn4pmgw and is included for printing as supporting information file 1 with this article.

## Expected results

**Fig 1:**
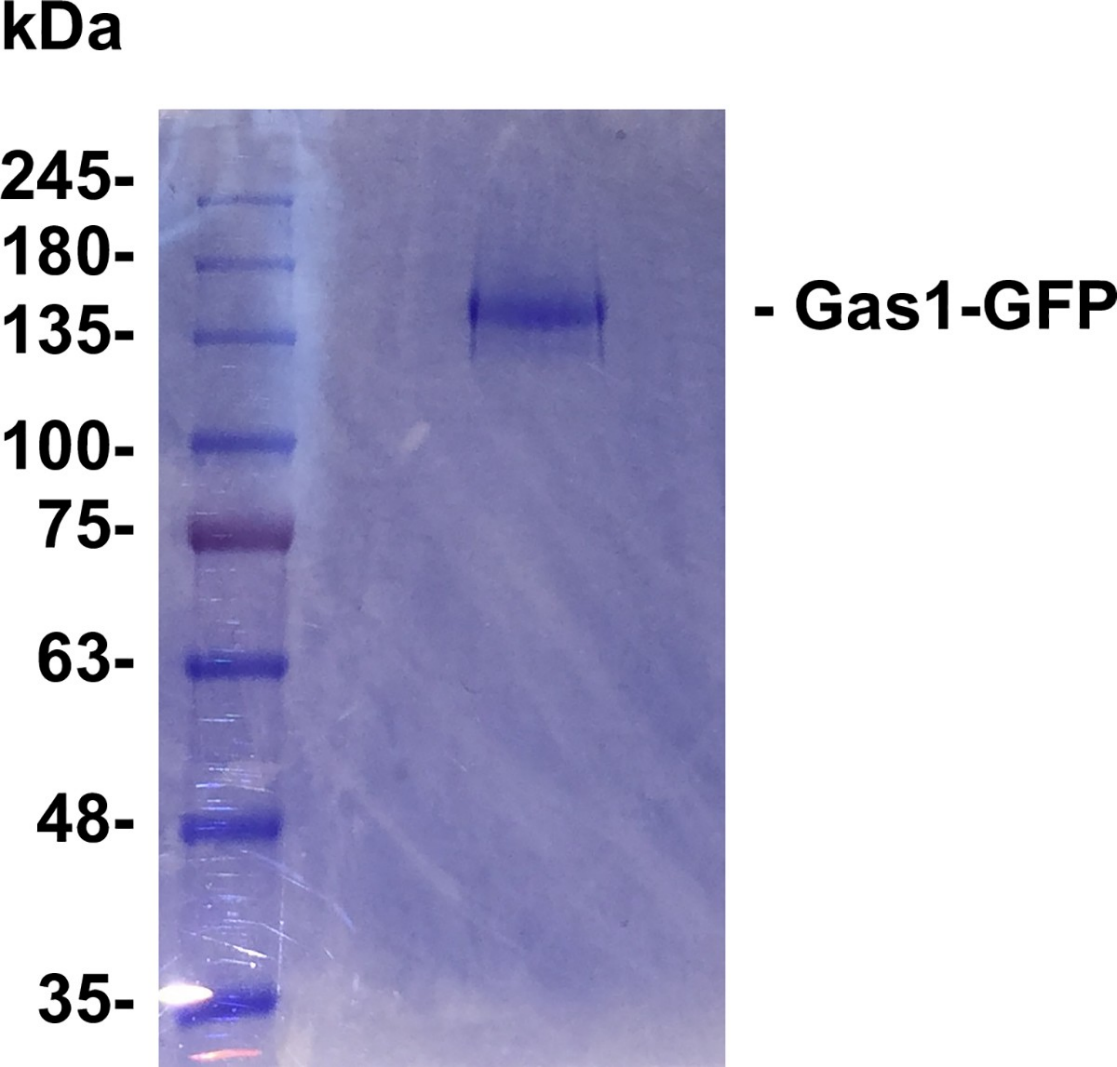
Coomassie Brilliant Blue stained SDS-electrophoresis gel of immunopurified Gas1-GFP. Gas1-GFP was constitutively expressed in wild type yeast cells, released by PI-PLC treatment from the cellular membrane fraction, immunopurified from the supernatant with GFP-Trap_A (ChromoTek) and electrophoresed by SDS-PAGE. The staining of the SDS-PAGE gel with Coomassie Brilliant Blue revealed only a band of 160 kDa, which corresponds to the plasma membrane form of Gas1-GFP. This stained band was excised from the gel and the Gas1-GFP protein in the gel slice was extracted for trypsin digestion and subsequent MS analysis of the GPI glycan.

In yeast, GPI-APs are required for cell wall biogenesis, morphogenesis and adhesion, being some of them retained at the plasma membrane and many others transferred to the cell wall [12]. The common precursor structure of the GPI glycan synthesized in the ER is Man-(EtNP)Man-(EtNP)Man-(EtNP)Man-GlcN. The remodeling of the GPI glycan during GPI-AP transport from the ER to the cell surface can involve the addition of a 5^th^ Man on the 4^th^ Man by unidentified enzymes, and the removal of the side-branch EtNP of Man1 and Man2 by the phosphodiesterase enzymes Cdc1 and Ted1 respectively [3,13,14]. We were interested to elucidate the specific remodeled GPI glycan structure of the plasma membrane protein Gas1 tagged with GFP. For this purpose, Gas1-GFP was constitutively expressed in a yeast wild type strain, enzymatically released from the cellular membrane fraction, immunopurified from the supernatant and electrophoresed by SDS-PAGE. After staining the SDS-PAGE gel with coomasie blue, only a stained band of 160 kDa was visible, which corresponds to the mature form of Gas1-GFP present at the plasma membrane (Fig 1). The Gas1-GFP protein was extracted from previously excised gel band and analyzed by MS after trypsin digestion.

**Fig 2:**
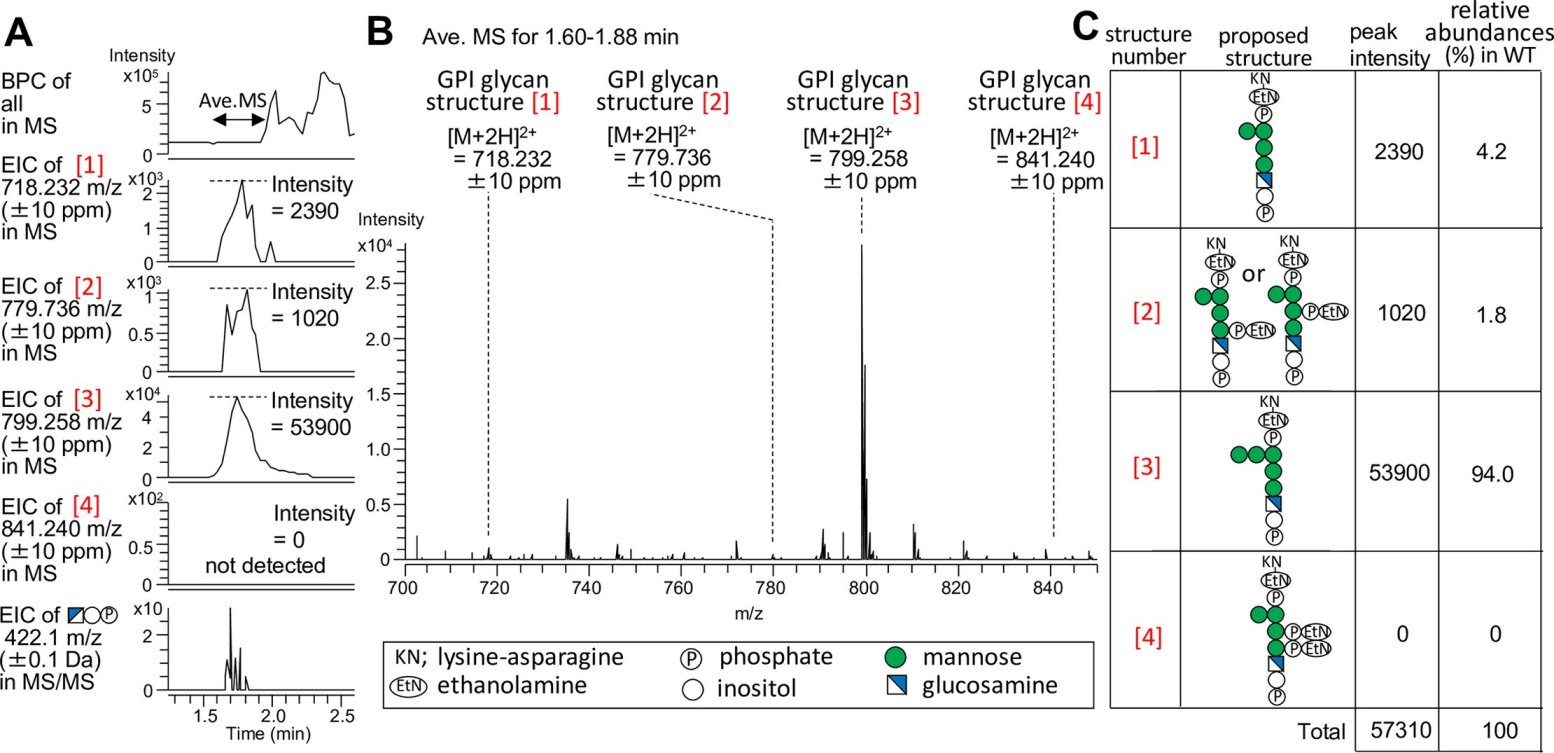
MS analysis of remodeled GPI-glycan structure of Gas1-GFP. (A) The base peak chromatogram (BPC) and the extracted ion chromatograms (EICs) of GPI glycan found in Gas1-GFP immunoprecipitated from wild-type. The bottom panel is EIC of a GPI-specific fragment ion treated with PI-PLC (m/z 422.1, GlcN-Ino-P) in MS/MS. (B) The averaged mass spectra (Ave. MS) for 1.60 to 1.88 min on BPC. (C) The proposed structures and the relative abundances (%) of the GPI glycan found in Gas1-GFP immunoprecipitated from wild-type. The relative abundance (%) of each GPI glycan was calculated from the peak intensity of monoisotopic masses of each GPI glycan in EIC using Xcalibur software ver. 2.2. (Thermo Fisher Scientific). These structures contain GPI peptides (KN) bearing hexosamine (glucosamine) attached to Man1 as reported previously [6].

**Fig 3:**
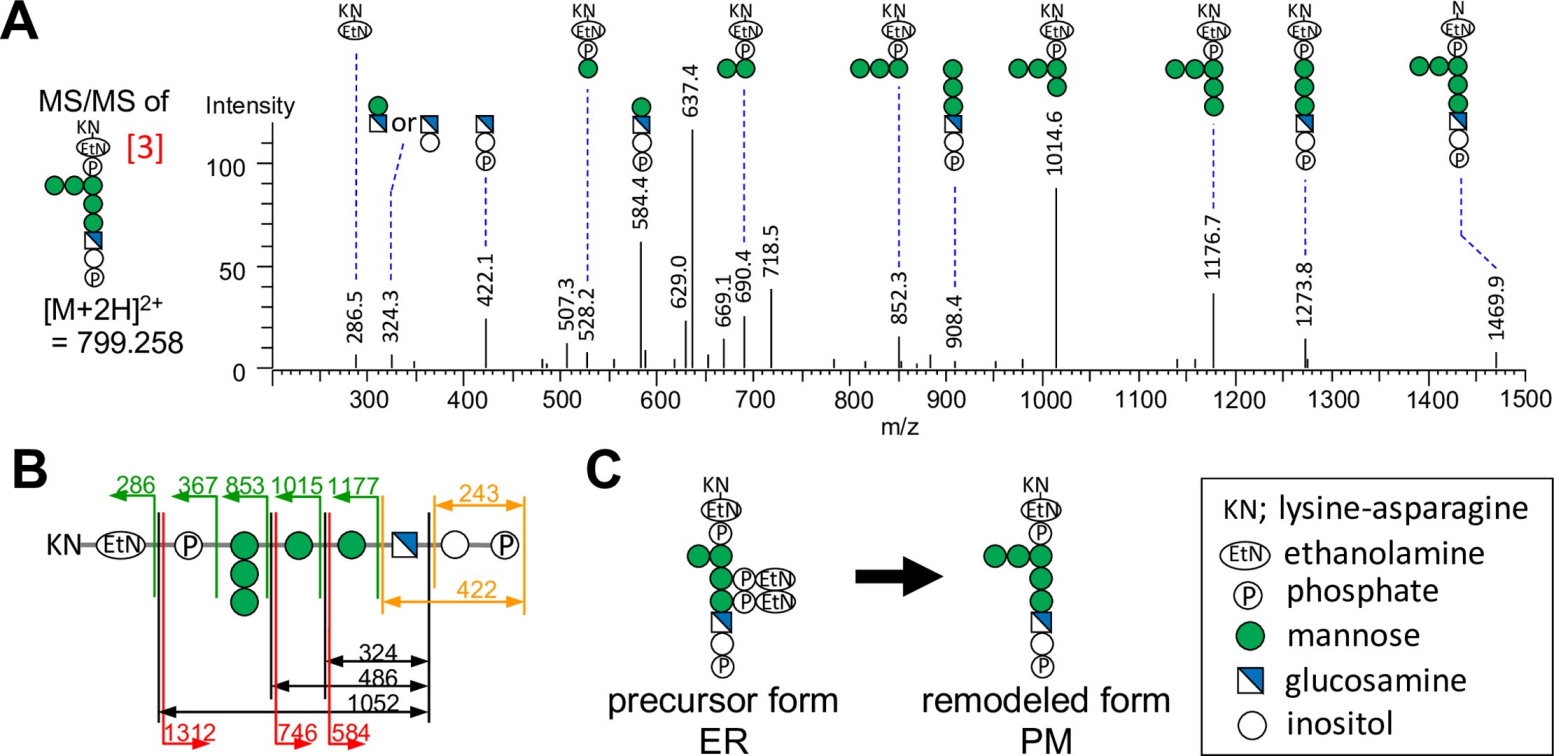
MS/MS analysis of remodeled GPI-glycan structure of Gas1-GFP. (A) MS/MS spectra of GPI glycan [3] found in Gas1-GFP immunoprecipitated from wild-type. Each fragment ions are shown with the deduced structures and the observed mass. (B) The theoretical fragmentation pattern of GPI glycan [3] found in Gas1-GFP immunoprecipitated from wild-type. The b-series fragments are indicated by green arrows, and the terminal and internal fragments of the GPI structure are indicated by red and black arrows, respectively. The orange arrows indicate m/z 422 and 243, a GPI-specific fragment ion treated with PI-PLC [6]. (C) Scheme of GPI glycan remodeling of Gas1-GFP. The remodeling of the GPI glycan during transport of Gas1-GFP from the ER to the plasma membrane (PM) involves the addition of a 5th Man on the 4th Man and the removal of the side-branch EtNP of Man1 and Man2.

The base peak chromatogram (BPC) of GPI glycan by MS analysis is shown in the top panel of Fig 2A. The extracted ion chromatograms (EICs) of the peptides containing GPI-glycans using their theoretical mass of the proposed structures are shown in the second to fifth panels of Fig 2A. The bottom panel of Fig 2A is EIC of a GPI-specific fragment ion treated with PI-PLC (m/z 422.1, GlcN-Ino-P) in MS/MS. We found out the peptides containing GPI-glycans using both EICs of their peptides in MS and EIC of the fragment ion in MS/MS. In the Gas1-GFP sample, peptides bearing GPI glycans were observed in the pass-through fraction (1.60–1.88 minutes), because the peptides were very short with the two amino acids, which is lysine-asparagine (KN). In theory, the peptide should have been composed only of N after trypsin digestion, but steric hindrance may have made it difficult to cut between K and N. N bearing GPI glycan was slightly observed at about the same retention time as KN bearing GPI glycan, but we used the major KN bearing GPI glycans for the qualitative and relative quantification of this study. Therefore, the averaged MS of that time (1.60–1.88 minutes) was performed, and the result is shown in Fig 2B. The three proposed structures of GPI glycans [1–3] shown in Fig 2C were detected as ion peaks at m/z 718.232, 779.736 and 799.258 of [M+2H]^2+^, respectively. As expected, the proposed structure of GPI glycan [4], corresponding to the initial ER precursor form of the GPI glycan, was not detected in the wild type sample. The relative abundance (%) of each GPI glycan was calculated from the peak intensity of monoisotopic masses of each GPI glycan in the extracted ion chromatogram (EIC) shown in second -fourth panels of Fig 2A. Next, the MS/MS analysis of peptides bearing GPI glycans of [3] was conducted. In Fig 3A, the detection of fragment ions allowed the identification of GPI glycan of structure [3]. The GPI glycan of structure [1] and [2] were not subjected to acquisition of MS/MS data due to tiny amount. The theoretical fragmentation pattern of GPI glycan [3] found in Gas1-GFP is shown in Fig 3B. Most of the peptides bearing GPI (94%) consisted of KN-EtNP-(Man4-Man5)-Man3-Man2-Man1-GlcN-Ino-P (*m/z*799.258) in wild type cells (Fig 2C, [3]). Therefore, our result indicates that remodeling of the GPI glycan of Gas1-GFP involves the addition of a new Man to Man4, and the removal of the EtNPs from Man1 and Man2 by the phosphodiesterase enzymes Cdc1 and Ted1 respectively (Fig 3C).

## Supporting information

S1 File Step-by-step protocol, also available on protocols.io. (PDF)

S1 Raw images (TIF)
