# Supplementary material for: Structural analysis of the GPI glycan
Source: PLoS One. 2021 Sep 16;16(9):e0257435. doi: 10.1371/journal.pone.0257435 (PMC8445438; doi:10.1371/journal.pone.0257435)
Supplement: S1 File — (PDF) [file pone.0257435.s001.pdf]

Structural analysis of the GPI glycan

Miyako Nakano †[1], Susana Sabido-Bozo †[2], Kouta Okazaki[3], Auxiliadora Aguilera-Romero[2], Sofia Rodriguez-Gallardo[2], Alejandro Cortes-Gomez[2], Sergio Lopez[2], Atsuko Ikeda[1], Kouichi Funato[1], Manuel Muñiz[2]

[1]Graduate School of Integrated Sciences for Life, Hiroshima University, Higashi-Hiroshima, Hiroshima 739-8528, Japan;
[2]Departamento de Biologia Celular, Universidad de Sevilla e Instituto de Biomedicina de Sevilla (IBiS), Hospital Universitario Virgen del Rocío/CSIC/Universidad de Sevilla, 41012, Sevilla, Spain;
[3]Graduate School of Integrated Sciences for Life, Hiroshima University, Higashi-Hiroshima, Hiroshima 739-8528, Japan.

1 *Works for me*

Manuel Muniz

ABSTRACT

Glycosylphosphatidylinositol (GPI) anchoring of proteins is an essential post-translational modification in all eukaryotes that occurs at the endoplasmic reticulum (ER) and serves to deliver GPI-anchored proteins (GPI-APs) to the cell surface where they play a wide variety of vital physiological roles. This paper describes an optimized method for purification and structural analysis of the GPI glycan of individual GPI-APs in yeast. The protocol involves the expression of a specific GPI-AP tagged with GFP, enzymatic release from the cellular membrane fraction, immunopurification, separation by electrophoresis, and analysis by mass spectrometry after trypsin digestion. We used specifically this protocol to address the structural remodeling that undergoes the GPI glycan of a specific GPI-AP during its transport to the cell surface. This method can be also applied to investigate the GPI-AP biosynthetic pathway and to directly confirm the predicted GPI-anchoring of individual proteins.

PROTOCOL INFO

Miyako Nakano †, Susana Sabido-Bozo †, Kouta Okazaki, Auxiliadora Aguilera-Romero, Sofia Rodriguez-Gallardo, Alejandro Cortes-Gomez, Sergio Lopez, Atsuko Ikeda, Kouichi Funato, Manuel Muñiz . Structuralanalysis of the GPI glycan. **protocols.io**
https://protocols.io/view/structural-analysis-of-the-gpi-glycan-bxn4pmgw

KEYWORDS

GPI glycan, GPI anchored-proteins, Mass spectrometry

CREATED

Aug 24, 2021

LAST MODIFIED

Aug 24, 2021

PROTOCOL INTEGER ID

52668

MATERIAL TEXTS

Recipes

Growth media

1. SD agar plates: synthetical minimal medium containing 2% glucose, 0.67% yeast nitrogen base, 0.5% ammonium sulfate, and 2% bacteriological agar, supplemented with the appropriate nutritional requirements (0.012% except for adenine 0,03%) to complement auxotrophies.
2. SD medium: synthetical minimal medium containing 2% glucose, 0.67% yeast nitrogen base and 0.5% ammonium sulphate, supplemented with the appropriate nutritional requirements (0.012% except for adenine 0,03%) to complement auxotrophies.
3. YPD medium: rich medium containing 2% glucose, 1% yeast extract, and 2% peptone, supplemented with 0.2% adenine and uracil.

Buffers

1. 1000x PI (Protease inhibitor cocktail): antipain 0.1%, leupeptin 0.1% and pepstatin 0.1% in DMSO

2. 100x PMSF: 100mM phenylmethylsulphonyl fluoride in isopropanol
3. TNE: 50 mM Tris-HCl, pH 7.5, 150 mM NaCl, 5 mM EDTA, 1 mM PMSF, 1x PI
4. TNE-D 0,2%: TNE containing 0,2% (w/v) digitonin
7. Stock of Ammonium acetate 1M (MS grade) in water (MS grade)
8. Destaining solution: Isopropanol 20%, Acetic acid 10%
7: SB 2X (Sample buffer): 4% SDS, 20% glycerol, 10% 2-mercaptoethanol, 0.004% bromophenol blue and 0.125 M Tris HCl, pH 6.8
9. Coomasie Brilliant Blue (CBB)
10. destaining solution: 30% CH_3CN, 50 mM NH_4HCO_3
11. reduction solution: 10 mM dithiothreitol (DTT) and 10 mM ethylenediamine tetra-acetic acid (EDTA) in 50 mM NH_4HCO_3 Prepare just before use
12. alkylation solution: 40 mM iodoacetamide (IAA) and 10 mM EDTA in 50 mM NH_4HCO_3. Prepare just before use
13. trypsin solution: 20-100 ng/μL of trypsin in 50 mM NH_4HCO_3. Prepare just before use
14. extraction solution 1: 50% CH_3CN/0.1% (v/v) trifluoro acetic acid (TFA) in ultrapure water
15. extraction solution 2: 10% CH_3CN/0.1% (v/v) trifluoro acetic acid (TFA) in ultrapure water
16. solvent A of LC-ESI MS: 0.08% formic acid in ultrapure water
17. solvent B of LC-ESI MS: 0.15% formic acid in 80% acetonitrile

Equipment
1. Incubator shaker (Eppendorf, model M1299-0092)
2. Micropipettes (Gilson, Pipetman, models: P20, P200, P1000)
3. Spectrophotometer or microplate reader (any brand with 600 nm wavelength) or equipment to count cell density
4. Bead beater (Fastprep, MP Biomedicals, model: Fastprep-24).
5. Centrifuge (Thermo scientific, model: Heraeus Fresco 17; Eppendorf, model: centrifuge 5818R)
6. Orbital wheel (any brand)
7. Electrophoresis system (Mini PROTEAN, Bio-Rad catalog number: 1658005)
8. Power supply (Bio-Rad, catalog number: 1645052)
9. Bio Shaker (Taitec, model: M-BR-022UP)
10. Shaker (Twin Mixer, AS-ONE, model: TM-282)
11. Ultrasonicator (Ultrasonic Cleaner, AS-ONE, model: MCS-3)
12. Vacuum Concentrators System (Speed Vac):
    a. Centrifugal Concentrator (TOMY, model: CC-105)
    b. Low-Temperature Trap (TOMY, model: TU-500)
    c. Oil Rotary Vacuum Pump (PHIL, model: BSW-50)
13. Centrifuge (KUBOTA, model: 3740)
14. Liquid Chromatogram (LC)-Electro Spray Ionization (ESI) Mass Spectrometry (MS) System:
    a. LC system (Thermo Fisher Scientific, model: Accela)
    b. MS system (Thermo Fisher Scientific, model: LTQ Orbitrap XL (hybrid linear ion trap-orbitrap mass spectrometer))
15. ODS column (Nomura Chemicals, Develosil 300ODS-HG-5; 150x1.0 mm ID)

Materials
1. Budding yeast cells (W303) expressing Gas1-GFP
2. Toothpicks sterilized before use (any brand)
3. Yeast extract (Pronadisa catalog number: 1702.00)
4. Peptone (Panreac catalog number: A2210,0500)
5. Glucose (Panreac catalog number: 131341.0914)
6. Yeast nitrogen base without amino acids (Difco Laboratories, catalog number: 291940)
7. Nutritional supplements:
    a. Uracil SIGMA catalog number: U0750
    b. L-Leucine SIGMA catalog number: L8000
    c. L-Tryptophan SIGMA catalog number: T0254
    d. Adenine SIGMA catalog number: A8626
    e. L-Histidine SIGMA catalog number: H8000
    f. L(+)-Lysine SIGMA catalog number: L5501
    g. L-Methionine SIGMA catalog number: M9625)
8. Agar (Oxoid catalog number: LP0011)
9. 50 ml Falcon tubes (any brand)
10. 1,5 ml screw-cap microcentrifuge tubes (any brand)
11. Glass beads (SIGMA catalog number: G9268-500G)
12. 1,5 ml UltraClear microcentrifuge tubes (Axygen catalog number: MCT-175-L-C)
13. Digitonin (Panreac AppliChem catalog number: A1905,0005)
14. GFP-Trap Agarose beads (ChromoTek, catalog number: gta-20)
15. Protease Inhibitors (PI):
    a. Antipain (SIGMA catalog number: A6191)
    b. Leupeptin (SIGMA catalog number: L2023)

c. Pepstatin A (SIGMA catalog number: P4265)

18. DMSO (Sigma catalog number: 154938-100ML)
19. PMSF (Amresco catalog number: 0754-5G)
20. Isopropanol (Panreac catalog number: 131090.1211)
21. Tris (Amresco catalog number: 0497-1KG)
22. NaCl (Panreac catalog number: 131659.1211)
23. EDTA (Amresco catalog number: 0322-500G)
24. HCl 5mol/l (Panreac catalog number: 182109.1211)
25. Acetic Acid Glacial (Panreac catalog number: 141008.1211)
26. 7.5 % Mini-PROTEAN® TGX™ Precast Protein Gels, 10-well, 30 µl (Bio-Rad Laboratories; 4561023)
27. 1,5 ml polypropylene microcentrifuge tube (Eppendorf Safe-Lock Tube, #0030 120.086)
28. Ammonium bicarbonate (NH4HCO3) (for Proteomics, FUJIFILM Wako Chemicals, #012-21745)
29. Acetonitrile (CH3CN) (for LC/MS, FUJIFILM Wako Chemicals, #018-19853)
30. Dithiothreitol (DTT) (for Molecular Biology, FUJIFILM Wako Chemicals, #048-29224)
31. Iodoacetamide (IAA) (for Proteomics, FUJIFILM Wako Chemicals, #099-05591)
32. Trypsin protease (MS grade, Thermo Fisher Scientific, #90057)
33. Vial for Accela autosampler (vial and cap; Thermo Fisher Scientific, #C4011-11 and #C4011-55)

Yeast growth and culture

1 Pick up the yeast cells expressing the plasma membrane GPI-AP Gas1-GFP from a frozen stock using a sterile toothpick, streak them on an SD medium without leucine (SD-LEU) agar plate and incubate them at 24 ºC for 2-3 days.

2 Inoculate the yeast cells into 4 ml SD-LEU medium and growth them for 6-8 h to the early-to-mid logarithmic phase at 24 ºC with shaking at 500 rpm.

3 Dilute yeast cells into 600 - 800 ml YPD medium and growth them to mid-log phase overnight at 24 ºC with shaking at 500 rpm.

4 On the next day, harvest 600 x 10^7 cells by centrifugation at 3000 x g for 5 min, discard the supernatant, resuspend the pellet in 12 ml of TNE precooled at 4 ºC and distribute aliquots of 1 ml into 2 ml screw-cap microcentrifuge tubes (12 tubes).

5 Centrifuge aliquots at 13000 x g for 1 min at 4 ºC, discard supernatants, and freeze the cell pellets at -80 ºC.

Cellular membrane fractionation

6 Quickly thaw the cell pellets and immediately place the tubes on ice.

7 Resuspend each cell pellet with 700 µl of prechilled TNE.

8 Add 600 µl of glass beads per tube.

9 Lyse mechanically the cells using a bead beater system. For Fastprep device: 3 cycles of 20 sec at 5 m/s. Rest on ice for 3 min between cycles.

10 Spin down beads and cells debris at 1000 x g for 10 min at 4 ºC and transfer supernatants into 1,5 ml UltraClear

microcentrifuge tubes from Axygen (UC tube) (item 12 from the list of materials).

11 Spin down cellular membranes at 17000 x g for 1h at 4 ºC.

12 Discard the supernatant and resuspend each membrane pellet in 100 µl of TNE.

13 Collect the resuspended membrane pellets into two UC tubes.

14 Spin down the membrane pellets at 17000 x g for 1h 4 ºC.

PI-PLC treatment

15 Resuspend each membrane pellet in 440 µl TNE and collect them into a UC tube.

16 Add 10 µl of PI-PLC (Invitrogen 0,1U/µl) and 50 µl TNE-D 0,2%.

17 Incubate at 37 ºC for 1h.

18 Spin down the membranes at 17000 x g for 30min at 4 ºC.

19 Discard the membrane pellet and save the supernatant into a UC tube at 4 ºC.

Immunoprecipitation (IP)

20 Prepare 30% GFP-Trap_A beads by vortexing to remove aggregates then washing in TNE twice before resuspending in TNE to a concentration of 30%.

21 Add 100 µl of GFP-Trap beads slurry into a new UC tube (IP tube).

22 Add the 450 µl of the previously saved supernatant (step 19) to the IP tube from step 21.

23 Incubate the IP tube rotating at 4 ºC overnight.

24 The next day, centrifuge the IP tube at 5000 x g for 30 sec at 4 °C to spin down the beads and discard the supernatant.

25 Resuspend the beads pellet with 500 µl of TNE-D 0,2% and transfer to a new UC tube.

26 Wash the original IP tube from step 24 with 500 µl of TNE-D 0,2% and combine the beads suspension with the first one into the new UC tube from step 25.

27 Centrifuge at 5000 x g for 30 s at 4 °C and remove the supernatant.

28 Add 750 µl of TNE-D 0.2%. Repeat steps 27 and 28 three times more.

29 Centrifuge at 5000 x g for 30 s at 4 °C and remove the supernatant. Centrifuge again to remove the remaining liquid and dry the beads using a white micropipette tip.

30 Add 40 µl of sample buffer 2x.

31 Heat the samples at 95 °C for 5 min.

32 Vortex. Centrifuge maximum speed for 5 min at room temperature.

33 Load the supernatant onto a 7.5 % acrylamide gel and separate the immunoprecipitated Gas1-GFP by SDS-PAGE gel electrophoresis.

34 Stain the polyacrylamide gel with coomassie brilliant blue (CBB).

Preparation of the sample for mass spectrometry (MS) analysis

35 Excise a gel band (~2 mm × 8 mm) of protein stained with CBB from the polyacrylamide gel.

36 Cut the gel band into ~1 mm^3 pieces and put the gel pieces in a 1,5 ml polypropylene Safe Lock microcentrifuge tube from Eppendorf (P tube) (item 27 from the list of materials).

37 Add 200 µl of the destaining solution to the P tube.

38 Mix the sample on a shaker continuously for 20 min at room temperature. After 20 min of mixing, remove and discard the liquid using a pipette.

39 Repeat steps 37 and 38 one or two more times until removing CBB from gel pieces.

40 Add 200 µL of acetonitrile (CH3CN) to the gel pieces.

41 Mix the sample on a shaker continuously for 10 min at room temperature. After 10 min of mixing, remove and discard the liquid. The gel pieces will shrink and become opaque with this treatment.

42 Add 200 µL of the fresh reduction solution to the shrinking and opaque gel pieces in the sample P tube and incubate the P tube at 65 ºC for 1 hour. After 1 h incubation, remove and discard the liquid.

43 Add 200 µL of the fresh alkylation solution to the gel pieces in the sample P tube and incubate the P tube in the dark at room temperature for 30 min. After 30 min of incubation, remove and discard the liquid.

44 Add 200 µL of wash solution (50 mM NH4HCO3) to the sample P tube.

45 Mix the tube on a shaker continuously for 10 min at room temperature. After 10 min of mixing, remove and discard the liquid.

46 Repeat steps 44 and 45 one more time.

47 Add 200 µL of acetonitrile (CH3CN) to the gel pieces.

48 Mix the sample on a shaker continuously for 10 min at room temperature. After 10 min of mixing, remove and discard the liquid. The gel pieces will shrink and become opaque with this treatment.

49 Add trypsin solution to the sample P tube at 1/20–1/100 enzyme(ng): substrate(ng) with the minimum volume that the swelled gel can cover.

50 Preincubate the sample for 5 min at room temperature.

51 Add digestion solution (50 mM NH_4HCO_3) to the sample P tube to completely cover the gel pieces.

52 Incubate the sample P tube at 37 ºC overnight.

53 Suck and transfer the liquid to a new P tube using a pipette.

54 Add 200 µL of extraction solution 1 to the gel pieces.

55 Sonicate the sample P tube containing gel pieces for 10 min at room temperature using float.

56 Suck and transfer the liquid to the P tube from step 53 using a pipette.

57 Add 200 µL of extraction solution 2 to the gel pieces.

58 Sonicate the P tube containing gel pieces for 10 min at room temperature using float.

59 Suck and transfer the liquid to the P tube from step 53 using a pipette.

60 Dry the combined liquid in the P tube from 53 with a Speed Vac at room temperature for 2–4 hours (do not use heating). The dried samples can be stored in the freezer prior to LC-ESI MS analysis.

Mass spectrometry analysis of the peptides bearing GPI glycans

61 Dissolve the dried residue with 20 µL of ultrapure water for LC-ESI MS analysis.

62 Centrifuge at 13,200 x g for 5 min at 4°C and transfer 12 µL of the supernatant to a new vial for Accela autosampler.

63 Inject 8 µL of sample solution into LC.

Separate the peptides bearing GPI-glycans using an ODS column under specific gradient conditions.

64

65 Elute the column with solvent A for 5 min, at which point increases the concentration of solvent B to 40 % over 55 min at a flow rate of 50 µL/min using LC system.

66 Introduce continuously the eluate into an ESI source.

67 Analyze the Peptides bearing GPI-glycans by LTQ Orbitrap XL.

68 Set in the MS setting the voltage of the capillary source at 4.5 kV, and maintain the temperature of the transfer capillary at 300 ºC.

69 Set the capillary voltage and tube lens voltage at 15 and 50 V, respectively.

70 Obtain MS data in positive ion mode over the mass range m/z 300 to m/z 3000 (resolution: 60000, mass accuracy: 5 ppm).

71 Obtain MS/MS data by ion trap in LTQ Orbitrap XL (data dependent top 3, CID).

72 Analyze the MS and MS/MS data to identify the structure of GPI-glycans (See Fig. 2 and 3 of the associated article).
