# Supplementary figures and images for: Structural analysis of the GPI glycan
Source: PLoS One. 2021 Sep 16;16(9):e0257435. doi: 10.1371/journal.pone.0257435 (PMC8445438; doi:10.1371/journal.pone.0257435)

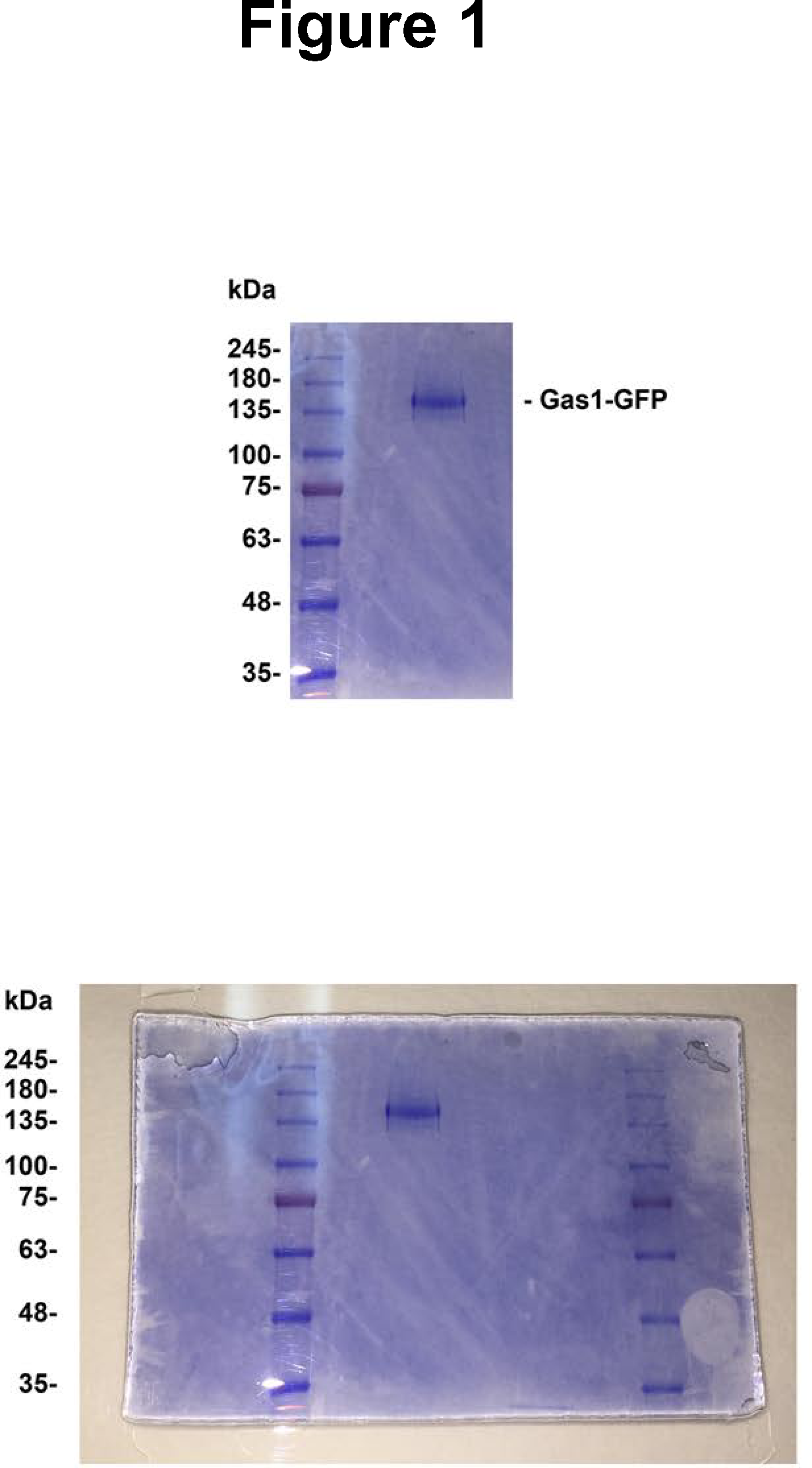

Supplement: S1 Raw images — (TIF) [file pone.0257435.s002.tif]
